# Environmental psychology as the science of context: grand challenges for the next decade

**DOI:** 10.3389/fpsyg.2026.1836854

**Published:** 2026-07-02

**Authors:** Francesco Ruotolo, Francesco Aletta

**Affiliations:** 1 Laboratory of Cognitive Science and Immersive Virtual Reality, Department of Psychology, University of Campania “L. Vanvitelli”, Caserta, Italy; 2 Institute for Environmental Design and Engineering, The Bartlett, University College London, London, United Kingdom

**Keywords:** behavior change, context, contextual influences, environmental psychology, human–environment interaction, psychological processes, sustainability, systems thinking

## Introduction

Why should we consider the environments in which we live so fundamental to addressing the major challenges of our time? Climate change, inequality, demographic aging, digital transformation and social fragmentation are often considered economic, technological or political problems. However, human responses to these challenges do not emerge in the abstract, but within their specific contexts.

We know that these contexts can include natural and built environments, sensory landscapes, institutional arrangements, and, increasingly, digital architectures. And it is precisely through these contextual cues that environments can shape attention, regulate stress, influence perceived control, identity, and even affect trust. Often, this happens outside of conscious awareness.

We believe that environmental psychology is in a privileged position to study these interactions between people and their environment. Although this discipline has broadened its scope, moving from pro-environmental behavior to regenerative environments and the latest environmental neuroscience and digital contexts, what we see today is excessive fragmentation. Therefore, the central challenge for the next decade should be theoretical integration: *clarifying how contextual conditions become psychological processes and how these processes scale to collective outcomes*.

**Figure 1:**
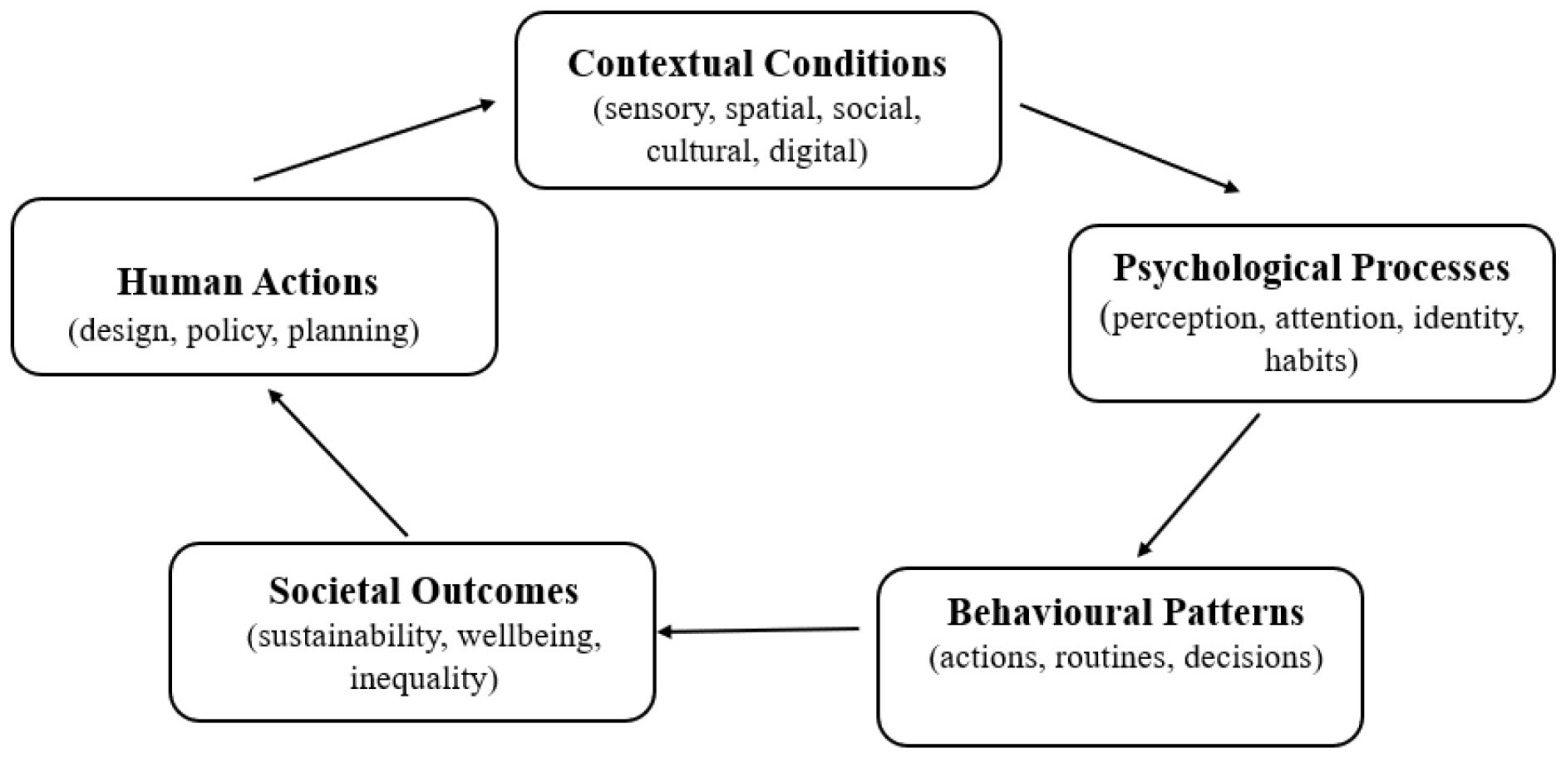
Environmental psychology as the science of context. Conceptual framework illustrating a dynamic feedback loop between contextual conditions, psychological processes, behavioral patterns, societal outcomes, and human actions.

We suggest that environmental psychology can be *productively reframed as a science of context*, i.e., a discipline that seeks to understand how sensory, spatial, social, cultural, and institutional conditions shape perception, behavior, wellbeing, and ultimately collective futures (see Figure 1).

## Why integration?

Environmental psychology has long been defined as the study of interactions between humans and their environment (e.g., Gifford, 2014). However, bibliometric and theoretical reviews have repeatedly highlighted its fragmentation and conceptual ambiguity (Giuliani and Scopelliti, 2009; Milfont and Page, 2013). Recent bibliometric evidence confirms this pattern. Analyzing over 4,300 articles published between 2004 and 2024, Ratcliffe et al. (2026) identified several thematic clusters ranging from human-nature relationships and attachment to place to pro-environmental behavior, climate change and virtual environments. Despite this vitality, there is still a lack of analysis capable of integrating these strands. As early as 1995, Stokols (1995) described this situation as a “paradox” of environmental psychology, highlighting how the discipline was united by a common interest in human-environment relationships, but often scattered in its conceptual boundaries.

We suggest that this integrative level lies in the concept of context. Across different research traditions, *environments are not merely objects of evaluation but conditions of human experience*. Understanding how contextual conditions shape psychological processes therefore could provide a common analytical framework linking otherwise separate domains of environmental psychology.

The limits of existing models become particularly visible in research on pro-environmental behavior. Meta-analytic evidence shows that traditional socio-psychological models explain behavioral intentions more successfully than actual behavior. (Bamberg and Möser 2007) estimated that intentions account for only about 27% of behavioral variance, while Kollmuss and Agyeman (2002) described the “value-action gap” between how much people care about the environment and the pro-environmental behaviors they actually engage in. However, dominant models focus primarily on internal variables such as attitudes, norms, and perceived control, underestimating the role of contextual structures and habitual action. For example, many environmentally relevant behaviors are often habitual and triggered by stable environmental stimuli (Verplanken and Wood, 2006). In addition, structural interventions, such as infrastructural changes or regulation, have been shown to often have stronger effects than informational strategies alone (Steg and Vlek, 2009). This suggests that the challenge should be not only motivational but also contextual. Rather than treating context as a background moderator, environmental psychology should conceptualize it as constitutive of psychological processes. Indeed, if we think about habits, we can clearly see that once a routine is established, behavior is triggered more by stable contextual stimuli than by deliberate intention. From this perspective, lasting behavioral change requires the transformation of the contextual architectures in which habits are embedded. So we could argue that environments do not simply constrain action, but actively participate in defining it.

## Mechanisms: how context becomes psychological

To function as an integrated discipline, environmental psychology must clarify the mechanisms through which environments exert influence. Three broad mechanisms can help explain this process: perceptual signaling, embodied regulation, and meaning-making.

### Environmental signals and perception

Environments constantly produce sensory and symbolic signals. Spatial configuration, density, noise, light, greenery, pollution, and digital interface design can modulate attention, cognitive load, and affective states. These signals can indicate possibilities for action (i.e., *affordances*, Gibson, 1977) and/or indicate what is salient and what is not. Furthermore, context is also represented by culture. Individuals are always embedded in layers of both place and culture (Tam and Milfont, 2020). Importantly, cultural norms, values, and institutional traditions shape how environmental signals are interpreted, which affordances are recognized, and which behaviors are considered legitimate or appropriate. Culture therefore operates as a constitutive dimension of environmental experience. Recent work in environmental neuroscience has begun to specify some of these pathways more precisely, especially by examining how measurable features of physical and social environments affect brain processes and behavior (Berman et al., 2019). For example, (Kühn and Gallinat 2024) have recently proposed that environments operate through identifiable “active ingredients” that are processed through particular sensory channels and neural mechanisms. These approaches are important because they help clarify how environmental signals are translated into embodied and neurocognitive processes, thereby contributing to a multilevel understanding of person–context transactions in environmental psychology.

### Embodied regulation

Exposure to the environment also influences physiological and neural processes. Environmental neuroscience has begun to show links between environmental properties and the regulation of stress and mental health (e.g., Kühn and Gallinat, 2024). This is consistent with what has previously been shown by environmental psychology: contact with restorative environments contributes to individuals' affective and physiological recovery (Hartig et al., 2014). However, previous analyses have highlighted the difficulty of the field in integrating molecular-level mechanisms with molar-level experience (Giuliani and Scopelliti, 2009). A central challenge is therefore to link embodied responses, such as stress physiology and attention restoration, with lived meaning and social interpretation. The development of such multilevel accounts remains essential for understanding how environmental exposure becomes psychologically and behaviorally relevant.

### Meaning, identity, and legitimacy

Environments not only regulate physiological states, but also communicate with the people who live in them. The way space is organized, its maintenance, its accessibility, its symbols, can indicate belonging or exclusion, fairness or abandonment. Through repeated exposure, individuals develop meanings associated with the place that shape their identity and attachment to it (Proshansky et al., 2014). Therefore, in order to address broader social dynamics, including social cohesion and democratic stability, it is important to understand how environments contribute to identity formation processes and perceptions of legitimacy. Although these links remain empirically underdeveloped, they indicate an important frontier for future interdisciplinary research.

In sum, all of the mechanisms mentioned above illustrate how contextual conditions can become psychological processes through perception, embodiment, and meaning-making.

## Scaling up: context and societal outcomes

The distinctive contribution of environmental psychology may lie in linking micro-level psychological processes to macro-level societal outcomes. Conceptual models of the human dimensions of climate change already reflect this multi-level logic. In fact, they link cognitive, affective, and motivational processes to human contributions, consequences, and responses such as mitigation and adaptation (Swim et al., 2011). However, a clear understanding of how context transforms individual experiences into social dynamics is still lacking.

### Sustainability as contextual transformation

Behavioral responses to climate change depend more on the contextual structures in which people live and act than on individual attitudes alone. For example, it has been shown that if the infrastructure, mobility systems, and social cues embedded in everyday environments are not changed, information campaigns alone will have limited effects (Verplanken and Wood, 2006; Steg and Vlek, 2009). In this sense, sustainability should be understood primarily as a process of transforming the contexts that organize behavior. In this regard, Bratman et al. (2019) have proposed considering exposure to nature as a “psychological ecosystem service,” highlighting how land use decisions can have direct effects on people's cognitive processes and affective states. At the same time, transitions to sustainability also involve place-based identities. Climate mitigation or adaptation strategies often require concrete changes to local landscapes and environments, for example through new energy infrastructure, changes in land use, or transformations of the territory. These changes can challenge the emotional relationship that people have with the place where they live and, for this reason, can generate resistance even among individuals who claim to be concerned about the environment (Devine-Wright, 2013).

### Inequality and environmental justice

Context is unevenly distributed. Access to green space, exposure to pollution, housing quality, and environmental amenities are systematically stratified by socioeconomic status (Bratman et al., 2019). Chronic exposure to adverse environments contributes to sustained stress, diminished perceived control, and reduced wellbeing. Inequality is therefore not only economic but spatially and environmentally experienced. Contextual asymmetries are not limited to territory. Institutional environments also structure behavior: in organizational contexts, perceived support and relational fairness influence environmental engagement, while breaches of the psychological contract undermine pro-environmental behavior (Paillé and Mejía-Morelos, 2014). Whether spatial or institutional, environments communicate reciprocity and legitimacy, shaping both wellbeing and action.

### Aging and lifespan contexts

Demographic aging intensifies the importance of person–environment fit. Environmental gerontology shows that spatial accessibility, walkability, and restorative environments influence autonomy, identity, and functional ability across the lifespan. Environmental exposure is also linked to health outcomes particularly relevant for aging populations (Hartig et al., 2014; Ruotolo et al., 2024). Therefore, contextual design contributes to shaping dignity and sustained agency. Converging evidence indicates that both direct contact with nature and dispositional connectedness to nature are independently associated with increased wellbeing and pro-environmental behavior (Capaldi et al., 2014; Martin et al., 2020). This suggests that context matters not only because individuals are exposed to environments, but also because environmental effects are shaped by how these environments are perceived and by individuals' psychological relationship with them.

### Digital and hybrid environments

The environments in which people live are increasingly hybrid, combining physical spaces with digitally mediated contexts. In the past, some authors had already called for the development of an environmental psychology of the Internet, anticipating the importance of these new contexts (Stokols and Montero, 2002). Today, the algorithmic systems embedded in digital platforms influence what people see, how they compare themselves to others, and which social norms appear most relevant. In this sense, digital infrastructures function as real environmental contexts. In fact, they organize attention, guide possibilities for action, and help shape identity and sense of agency, in a similar way to what happens in physical environments. Extending the analysis of context to digital systems is therefore an important frontier for environmental psychology.

Taken together, these examples show that people's behavior and wellbeing cannot be understood by looking at individuals alone. To understand human behavior, it is therefore necessary to study people and the contexts in which they live together, since psychological processes emerge through the interaction between individuals and their environments. From this perspective, environmental psychology can be understood as a discipline that analyses how contextual conditions shape psychological processes and behaviors within interdependent human–environment systems (Liu et al., 2007).

## Methodological and epistemological challenges

If environmental psychology is to operate as a science of context, methodological innovation is essential.

First, research should increasingly complement self-reported measures with observations of behavior *in situ*. Schultz and McCunn (2024) highlight the importance of moving beyond stated intentions toward measures of behavior in naturalistic contexts. For a discipline concerned with person–context transactions, understanding behavior requires examining how actions unfold within the environments where they occur. Importantly, these environments are not limited to physical settings: in an increasingly hybrid world, behavior also takes place within virtual environments, digitally mediated contexts and algorithmic architectures that structure attention, interaction, and decision-making.

Second, methodological pluralism should be embraced as integration rather than fragmentation. Qualitative and mixed methods provide indispensable insight into lived experience (Lloyd and Gifford, 2024), while physiological and neuroscientific approaches offer complementary evidence of embodied processes. The key challenge is achieving coherence across levels of analysis. Systems thinking provides one possible framework for integrating this disciplinary pluralism (Hogan and Weathers, 2003; Lezak and Thibodeau, 2016). Environmental psychology deals with complex adaptive systems in which causes and effects are distributed, delayed, and mutually reinforcing. Systems thinking helps make these dynamics explicit (Aletta et al., 2025), drawing on non-linearity paradigms, threshold effects, and emergent outcomes. This perspective aligns closely with environmental psychology's core commitment to people–environment transactions: environments shape behavior and experience, while human actions simultaneously reshape environments, altering subsequent perceptions, norms, and exposures.

Thirdly, the field must address its cultural limitations. For example, Tam and Milfont (2020) documented the predominance of Western, educated, industrialized, rich, and democratic (WEIRD) samples in environmental psychology. A discipline focused on context cannot remain contextually limited. From this perspective, cross-cultural research becomes essential. Recent initiatives also highlight the need to raise the academic level of the Global South and other structurally marginalized contexts through more equitable research and publication practices (Aruta and Whitmarsh, 2026).

Finally, for environmental psychology to develop as a cumulative science of context, it is necessary to insist on open science practices and transparent research procedures. This would allow research claims to be evaluated, reused, and extended to different studies and contexts (Nosek et al., 2015). In this regard, practices such as pre-registration, data sharing, and analytical transparency would facilitate comparison, reanalysis, and cumulative integration of results. Even more interestingly, reproducibility could be thought of as a systemic issue, i.e., linked to research incentives, reporting standards, and the efficiency with which knowledge is accumulated (Munafò et al., 2017). Strengthening open and reproducible practices is therefore essential for building robust and policy-relevant evidence.

## Conclusions

Since Barker's pioneering work (Barker, 1968) on behavioral environments, environmental psychology has recognized that human behavior is inseparable from the ecological contexts in which it develops. Environmental psychology should not be reduced to the study of nature, the built environment, sustainability or digital systems alone. Instead, its coherence may lie in the level of analysis: examining how contextual conditions shape psychological processes across different scales.

Humans are continually shaped by their environments. Environmental cues influence perception and stress regulation, just as repeated exposure contributes to identity and action, and aggregated contextual patterns shape social outcomes. Therefore, the great challenge for the next decade should be integration. In other words, we will need to link environmental cues to cognitive and neural mechanisms, connect individual psychological processes to structural conditions, and align disciplinary knowledge with social transformation.

We present this perspective as a heuristic for integration. Environmental psychology should not remain marginal to the study of social change, but rather offers a fundamental perspective for understanding how contextual conditions shape the future of humanity.
